# Classification of deep-sea cold seep bacteria by transformer combined with Raman spectroscopy

**DOI:** 10.1038/s41598-023-28730-w

**Published:** 2023-02-24

**Authors:** Bo Liu, Kunxiang Liu, Xiaoqing Qi, Weijia Zhang, Bei Li

**Affiliations:** 1 State Key Laboratory of Applied Optics, Changchun Institute of Optics, Fine Mechanics and Physics, Chinese Academy of Sciences, Changchun, 130033 People’s Republic of China; 2 University of Chinese Academy of Sciences, Beijing, 100049 People’s Republic of China; 3 Institute of Deep-Sea Science and Engineering, Chinese Academy of Sciences, Sanya, 572000 Hainan China

**Keywords:** Chemical biology, Microbiology

## Abstract

Raman spectroscopy is a rapid analysis method of biological samples without labeling and destruction. At present, the commonly used Raman spectrum classification models include CNN, RNN, etc. The transformer has not been used for Raman spectrum identification. This paper introduces a new method of transformer combined with Raman spectroscopy to identify deep-sea cold seep microorganisms at the single-cell level. We collected the Raman spectra of eight cold seep bacteria, each of which has at least 500 spectra for the training of transformer model. We compare the transformer classification model with other deep learning classification models. The experimental results show that this method can improve the accuracy of microbial classification. Our average isolation level accuracy is more than 97%.

## Introduction

Oceans occupy 70.8 percent of the earth's surface area. Cold seep is fluids from below the seabed sedimentary interface, which will overflow from the seabed in the form of leakage. The main components of these fluids are water, hydrocarbons (natural gas and oil), hydrogen sulfide and fine-grained sediments. These fluids are the source of various dense microbial and animal populations. Microbial populations in this unique environment have been extensively studied. Numerous studies have shown that deep-sea cold seeps, which contain large amounts of combustible ice resources, may be the third ecological environment on Earth found to carry out large-scale nitrogen fixation and provide large amounts of organic matter to deep-sea ecosystems. Therefore, it is very important to conduct research on deep cold seep bacteria and the identification and screening of specific functional bacteria^1^. The two main types of traditional methods of bacterial detection are bacteriological diagnosis and immunoserological diagnosis. Among them, bacteriological diagnosis is based on the morphology of bacteria (size, shape, arrangement, nucleoplasmic distribution, etc.), bacterial composition, metabolites and nucleic acids. The accuracy of morphological diagnosis is too low, while the study of bacterial composition and metabolism is often too costly, complicated and time-consuming. Immunoserological diagnosis, on the other hand, requires labeling of bacteria and expensive scientific instruments. Most of these methods require bacterial culture to complete microbial identification, delaying the detection process. Therefore, a new label-free, culture-free, non-contact, rapid bacterial identification method at the single-cell level is currently needed for bacteriological studies^2–4^. In recent years, Raman spectroscopy is often used in the rapid identification and analysis of microorganisms. Raman spectroscopy is an unlabeled^5–7^, non-invasive, rapid^8,9^ in situ cell identification method that can be used to identify^10^ and research^11^ microbial single-cell species. The single-cell Raman atlas of microorganisms contains a wealth of biochemical data in various physiological states.

Raman spectroscopy can provide biochemical information of bacteria, such as DNA, RNA, proteins, lipids, carbohydrates, etc. Raman spectra are equally capable of providing information about bacterial pigments^12–14^. For example, the Raman characteristic peaks of carotenoids, which are commonly found in microorganisms, are 1004, 1157, and 1520 cm^−1^ (C=C stretching vibration)^15^. In addition, Raman spectroscopy combined with machine learning, deep learning and other classification methods can reflect the differences between different species of microorganisms, thus enabling the identification of bacteria. Raman spectroscopy was able to distinguish Gram-positive from Gram-negative bacteria, and some peaks at 540 and 1380 cm^−1^ were significantly different for Gram-positive bacteria compared to Gram-negative bacteria^16^. Ho et al. successfully identified 30 common pathogens using deep learning, achieving an average separation level accuracy of over 82% and antibiotic treatment identification accuracy of 97.0 ± 0.3% on a low signal-to-noise spectrum^17^.

Current Raman spectral classification algorithms are classified into two types: feature-based classifiers and end-to-end deep learning classifiers. Typically, feature-based approaches derive numerical characteristics from raw signal data^18^. Partial least squares (PLS), principal component analysis (PCA), independent principal component analysis (ICA), and wavelet analysis (WA), among others, are signal processing algorithms used for feature extraction. The collected characteristics are then subjected to various classification methods based on multiple regression, such as linear discriminant analysis, support vector machine, random forest, and so on^19–21^. However, when sample sources and spectral acquisition conditions change, the spectral response to substances is not completely linear, which may reduce the model's prediction ability. Although support vector machines (SVM) are said to outperform most multivariate analysis methods^21^, their classification accuracy will be affected when dealing with large data sets. Traditional analytical modeling methods continue to perform poorly in terms of fit and robustness.

Deep learning-based classification methods have recently received a lot of attention because they perform well in certain classification tasks^22–25^. Deep learning method has powerful learning function and can eliminate the influence of nonlinearity. Convolutional neural networks (CNN) can accurately solve complex problems involving large amounts of data by simulating the structure and functions of computer biological neural networks^26^. However, CNN relies heavily on the choice of kernel. It will lose some time series information. If there is no deep structure, it is difficult to perceive the wide internal relationship of the signal, which may lead to a large number of calculations. Various recurrent neural networks (RNNs) have been proposed to learn the time characteristics of Raman signals^27^. However, the RNN steps cannot be parallelized and the efficiency is low. Another problem is that RNN only works on previous memory and current state. However, the Raman signal is continuous. Neither CNN nor RNN can well perceive the global dependence of Raman signals. In 2017, Google's machine translation team only adopted the attention mechanism to accomplish machine translation jobs, abandoning network architectures like RNN and CNN entirely^28^. Later, it was used in the field of image classification and achieved good results^29–32^. This method can calculate the representation of sequences with dependencies between different locations.

In this study, we build a Raman spectral database containing eight species of deep-sea cold seep bacteria and propose a new method for classifying Raman spectra using transformer structures. To the best of our knowledge, this is the first time that a transformer is used to classify Raman spectra. We use the commonly used classification methods and transformer structures to identify Raman spectra of deep-sea cold seep bacteria, and compare the identification results of different methods. The results show that the transformer structure obtained a high accuracy in the task of identifying eight species of cold seep bacteria.

## Materials and methods

### Sample preparation

We selected 8 pure cultured microorganisms of different species and genera isolated from deep-sea sediments. See Table S1 for the sea area where each microorganism is located. Select a single colony and transfer it to 10 ml 2216E liquid medium for activation. Inoculate into the fresh culture medium in the ratio of 1:100, culture at 150 rpm in a shaking table at 10 °C for 24 h, and then take 2 ml culture medium for centrifugation to collect the bacteria.

### Raman spectroscopy acquisition

**Figure 1:**
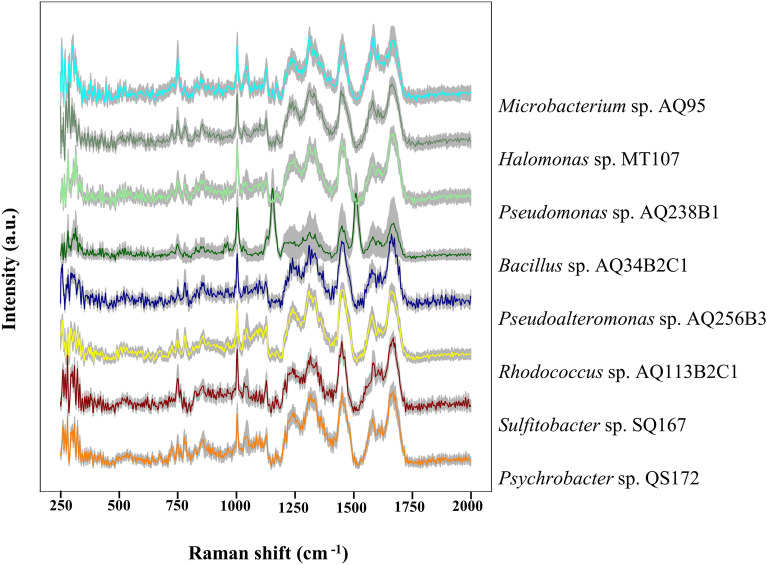
Raman spectra of eight cold seep microorganisms. Each strain has at least 500 spectra. The solid line represents the average value of Raman spectrum, and the standard deviation is represented by shadow.

In this experiment, the confocal Raman spectrometer (Hooke P300, Hooke Instrument Co., Ltd., China) is used to collect the Raman spectrum, equipped with 532 nm solid-state laser (Cobolt 08-DPL, Cobolt, Sweden) and—70 °C cooled CCD detector (PIXIS 100 B, Princeton instruments, USA). The laser is a continuous wave laser with an output power of 50mW, and the spectral bandwidth (FWHM) of the laser is 1 MHz. The laser beam was focused by a 100× objective (LMPlan FLN 100× , Olympus, Japan). The numerical aperture (NA) of the objective is 0.8, and the actual spot size after convergence by the objective is 406 nm.The power irradiated on each sample is 5mW and the exposure time is 5 s. The size of the bacteria was around 1 μm and we measured a Raman spectrum at the middle of each bacteria. At least 500 spectra were collected for each sample to limit the impact of spectral noise (Fig. 1).

### Data processing

**Figure 2:**
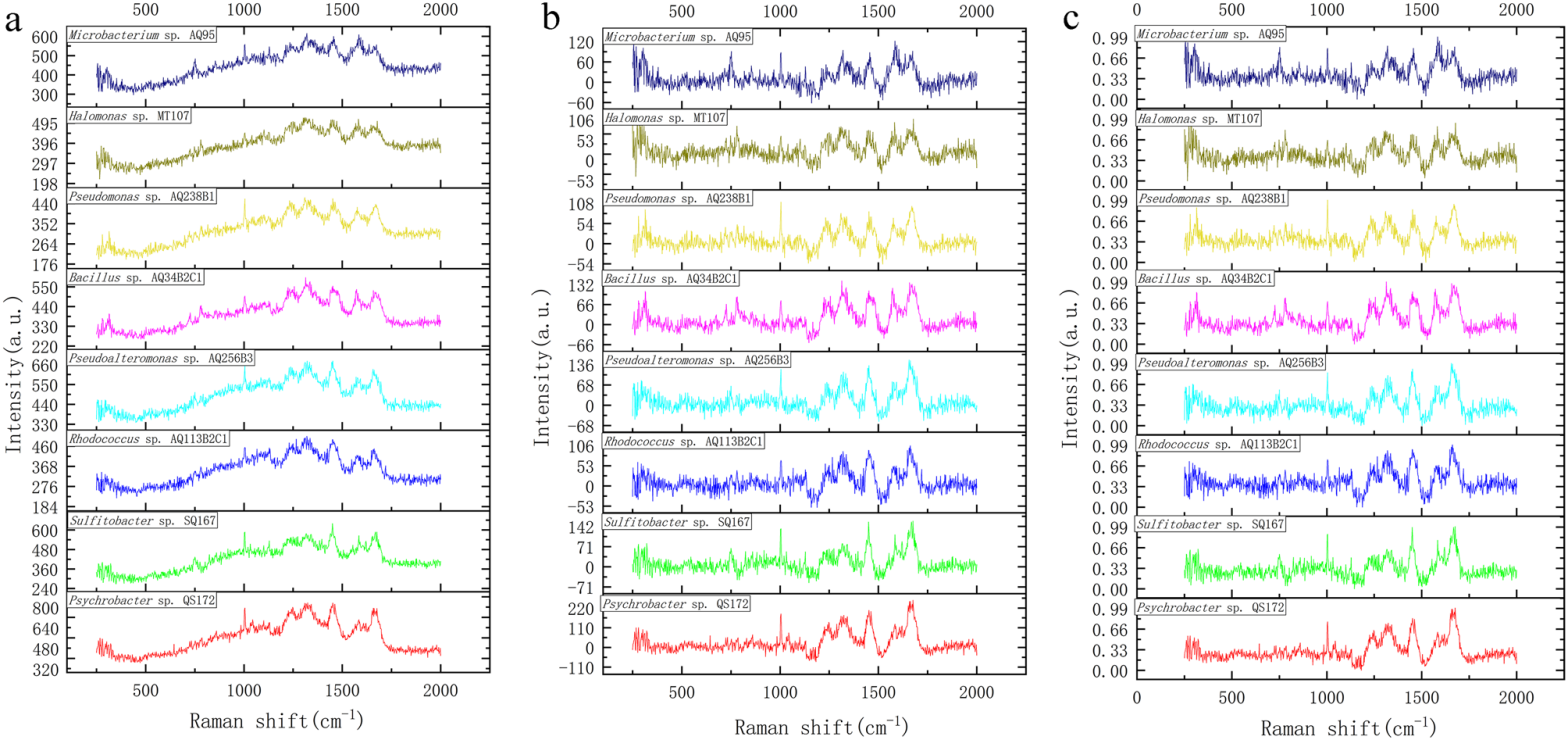
Examples of raw and preprocessed spectra examples. (a) Untreated spectra of 8 bacteria. (**b**) Spectra after baseline subtraction. (**c**) Spectra after normalization. These spectra are input into our model.

Each process from the transmitter to the receiver of the spectrometer may interfere with the noise of the obtained signal, which affects the further analysis of Raman spectrum. Therefore, it is very necessary to preprocess the collected spectral data. We removed cosmic rays from the spectrum, corrected the baseline with the *Subbackmod* function in Biodata's toolbox, and normalized with the *Mapminmax* function (Fig. 2)^33^.

## Results and discussion

### Model evaluation

In this study, we used AlexNet, ResNet models respectively to analyze and verify the feasibility and accuracy of Raman spectroscopy combined with deep learning model to classify cold-seep microorganisms. In order to better apply these methods to this experiment, we fine-tune the above model. For the above model, we use one-dimensional convolution layers instead of two-dimensional convolution layers.

We used the fivefold cross-validation method, to test the classification model of classifying data capabilities, and minimize caused by inappropriate dataset partition problems, such as the fitting model on the training set, the before-fitting results may not be a model, but because the dataset partition is not reasonable. First, we created five data sets by dividing the data for each bacterium into five equal portions. The classification model was divided into four groups for training, and one group was used as test data. In order to avoid over-fitting of the neural network, the four groups of data were randomly reorganized into two parts: 80% data for training and 20% data for verification. In the process of cross-validation, the accuracy of the five optimization models was compared, and the classification model with the best accuracy was selected from the five optimization models.

### Construction transformer network framework

**Figure 3:**
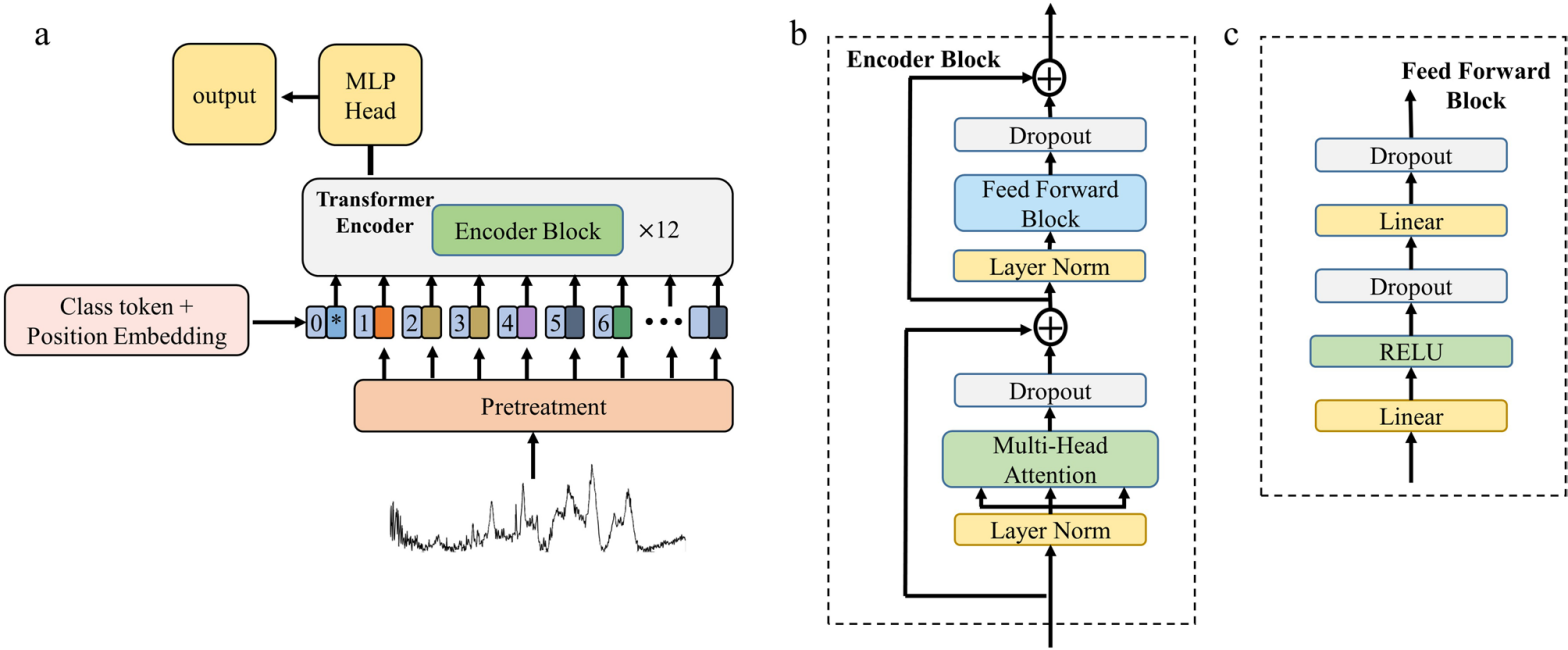
(**a**) Structure diagram of transformer classification model for Raman Spectrum Classification. (**b**) Structure diagram of encoder block. (**c**) Structure diagram of feedforward block.

Our network architecture is adapted from the description of Alexey dosovitskiy et al.^34^ as shown in Fig. 3a. The Raman spectrum is transmitted into the transformer model after pretreatment.。The transformer model is composed of a transformer encoder and a multi-layer perceptron (MLP), which is composed of a linear layer and an active layer. Position encoding is added to the input embedding at the top of the encoder to maintain the sequence's relative or absolute position. In addition, an extra learnable class token is fed to the transformer network that attends to all other tokens. The transformer encoder consists of 12 encoder blocks stacked repeatedly. Encoder block is composed of two blocks. The structure is shown in Fig. 3b. The first block is a multi-head attention block^28^, which uses 12 attention heads and an embedding dimension of 768. The second block is a simple fully connected feedforward neural network. The module framework of each fully connected feedforward neural network is shown in Fig. 3c. The two blocks are connected by the residual network structure. The Adam optimizer with default settings is used to train the model^35^. The cross-entropy function is used as the loss function.

### Classification results of cold seep bacteria by transformer model

**Figure 4:**
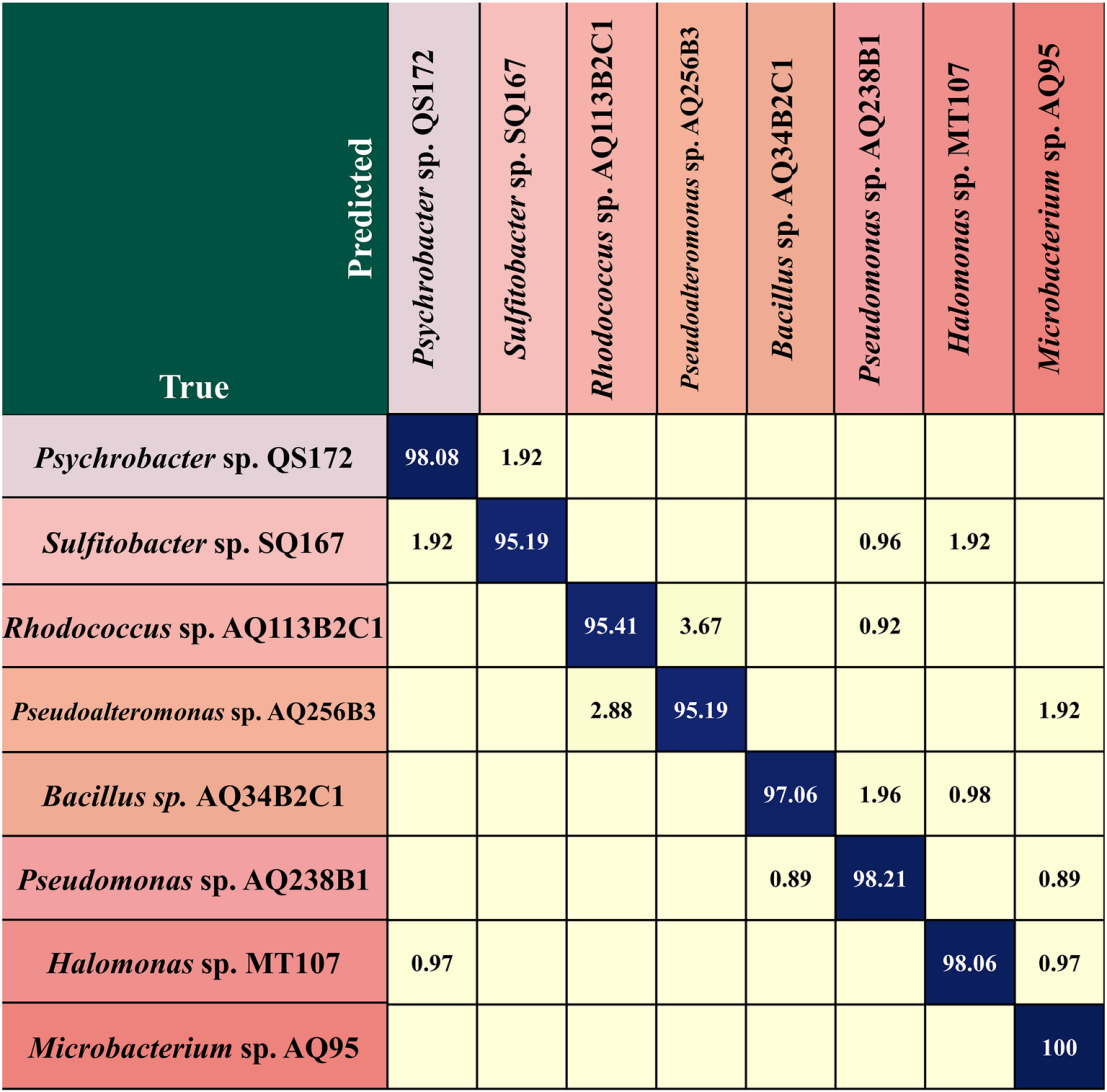
Confusion matrix of 8 cold seep bacteria. All spectra of each bacterium are classified into the correct category.

We used a trained transformer classification model to identify the species of each microbial cell based on the flora in the test data set. Each branching group in the test dataset was predicted by the trained Transformer classification model and assigned to a specific category。For identifying different microbial species, our transformer classification model has an average accuracy of 97.3%. As shown in Fig. 4, the classification accuracy of bacterial *Microbacterium* sp. AQ95 is 100%, the recognition accuracy of *Psychrobacter* sp. QS172, *Bacillus* sp. AQ34B2C1, *Pseudomonas* sp. AQ238B1 and *Halomonas* sp. MT107 is higher than 97% and the identification accuracy of *Sulfitobacter* sp. SQ167, *Rhodococcus* sp. AQ113B2C1, and *Pseudoalteromonas* sp. AQ256B3 is higher than 95%.

**Figure 5:**
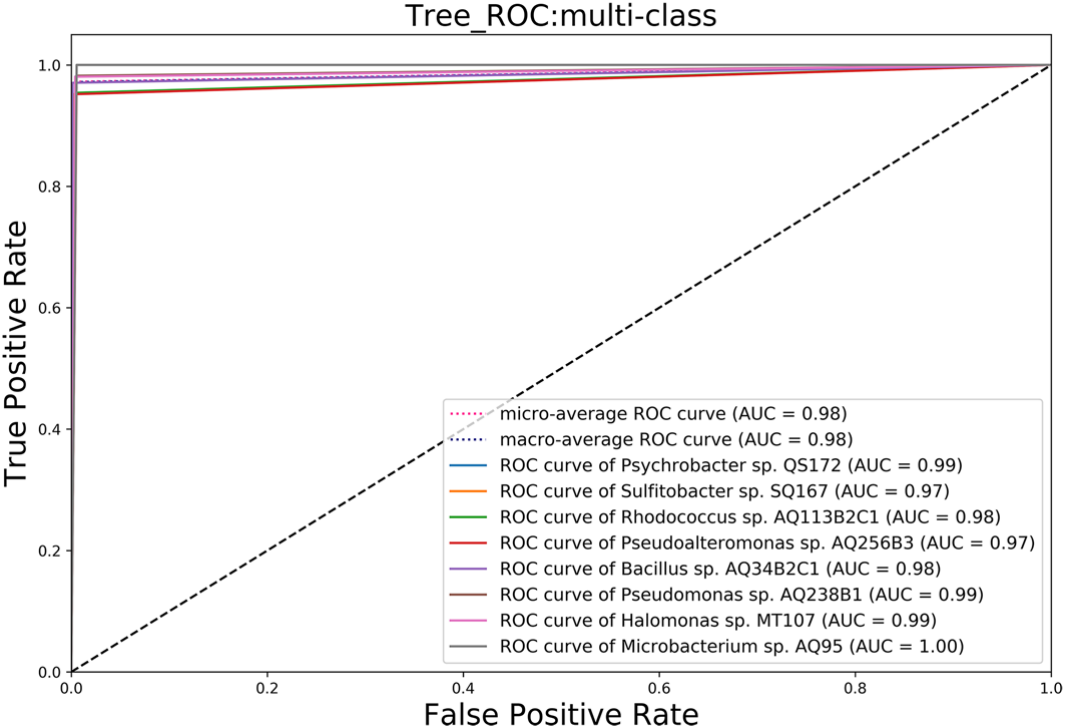
Receiver operating characteristic (ROC) curves of the Transformer model.

Receiver operating characteristic (ROC) curves were used to assess the specificity and sensitivity of five species classifications in the fivefold cross-validation study (Fig. 5). The eight strains' mean AUC (area under the ROC curve) was all greater than 0.97, indicating that our classification model had high specificity and sensitivity for classifying different microbial species.

In comparison, we used common analytical techniques such as deep learning to predict the types of single cells. We applied the original spectral data to AlexNet and ResNet for prediction, with the accuracy of 96.5% and 95.9% respectively. We plotted the confusion matrix of the above-mentioned classification model for deep-sea bacteria (Fig. S1, S2).

In conclusion, these results suggest that Raman spectroscopy combined with Transformer is a reliable method for the accurate identification of different microorganisms at the single-cell level.

## Conclusions

In this study, we analyzed eight kinds of microorganisms obtained from cold seep in different sea areas. We collected their Raman spectra and combined with transformer model to classify cold seep microorganisms. In addition, we used fivefold cross-validation to ensure good robustness of the model. Raman spectroscopy can be easily extended to new microbial applications due to its undamaging and unlabeled advantages. Meanwhile, the specificity, sensitivity, and accuracy of other common deep learning classification methods are compared. In addition, the new method of Raman spectrum classification proposed in this paper can also be applied to the accurate classification of other samples, which provides valuable insights for the accurate analysis of Raman data in the future.

## Supplementary Information

Supplementary Information.

## Data Availability

Data underlying the results presented in this paper are not publicly available at this time but may be obtained from the authors upon reasonable request.
