# Supplementary material for: Classification of deep-sea cold seep bacteria by transformer combined with Raman spectroscopy
Source: Sci Rep. 2023 Feb 24;13:3240. doi: 10.1038/s41598-023-28730-w (PMC9958026; doi:10.1038/s41598-023-28730-w)
Supplement: Supplementary file 1 — Supplementary Information. [file 41598_2023_28730_MOESM1_ESM.docx]

**Classification of deep-sea cold seep bacteria by Trans-former combined with Raman spectroscopy**

Bo Liu ^a, b†^, Kunxiang Liu ^a, b†^, Xiaoqing Qi ^c^, Weijia Zhang ^c*^, Bei Li ^a, b*^

^a^ State Key Laboratory of Applied Optics, Changchun Institute of Optics, Fine Mechanics and Physics, Chinese Academy of Sciences, Changchun 130033, P. R. China

^b^ University of Chinese Academy of Sciences, Beijing 100049, P. R. China

^c^Institute of Deep Sea Science and Engineering, Chinese Academy of Sciences, Sanya, Hainan 572000, China

*† These authors contributed equally to this work and should be considered co-first authors.*

^*^Correspondence: [beili@ciomp.ac.cn](mailto:beili@ciomp.ac.cn)

^*^Correspondence: wzhang@idsse.ac.cn

| Contents | Description |
| --- | --- |
| Supplementary Table 1 | The sea area where the strain is located |
| Supplementary Figure 1 | Classification accuracy results of AlexNet model |
| Supplementary Figure 2 | Classification accuracy results of ResNet model |

**Table S1** The sea area where the strain is located

| Sample number | Strain name | source |
| --- | --- | --- |
| *1* | *Psychrobacter* sp. QS172 | Northern South China Sea |
| *2* | *Sulfitobacter* sp. SQ167 | Hippocampal cold spring |
| *3* | *Rhodococcus* sp. AQ113B2C1 | Southwest Indian Ocean |
| *4* | *Pseudoalteromonas* sp. AQ256B3 | Southwest Indian Ocean |
| *5* | *Bacillus* sp. AQ34B2C1 | Southwest Indian Ocean |
| *6* | *Pseudomonas* sp. AQ238B1 | Southwest Indian Ocean |
| *7* | *Halomonas* sp. MT107 | Mariana Trench |
| *8* | *Microbacterium* sp. AQ95 | Southwest Indian Ocean |

b

A


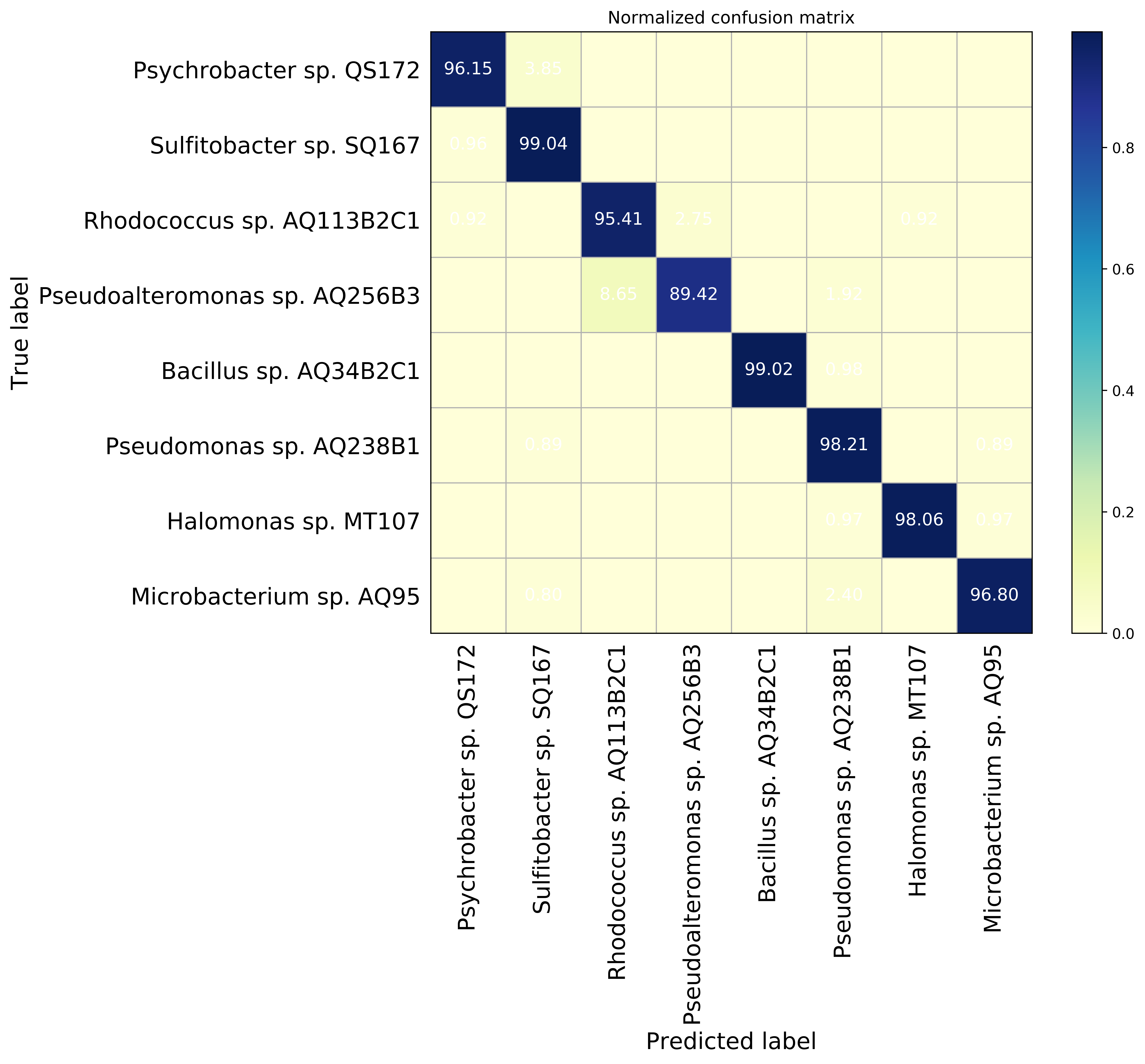

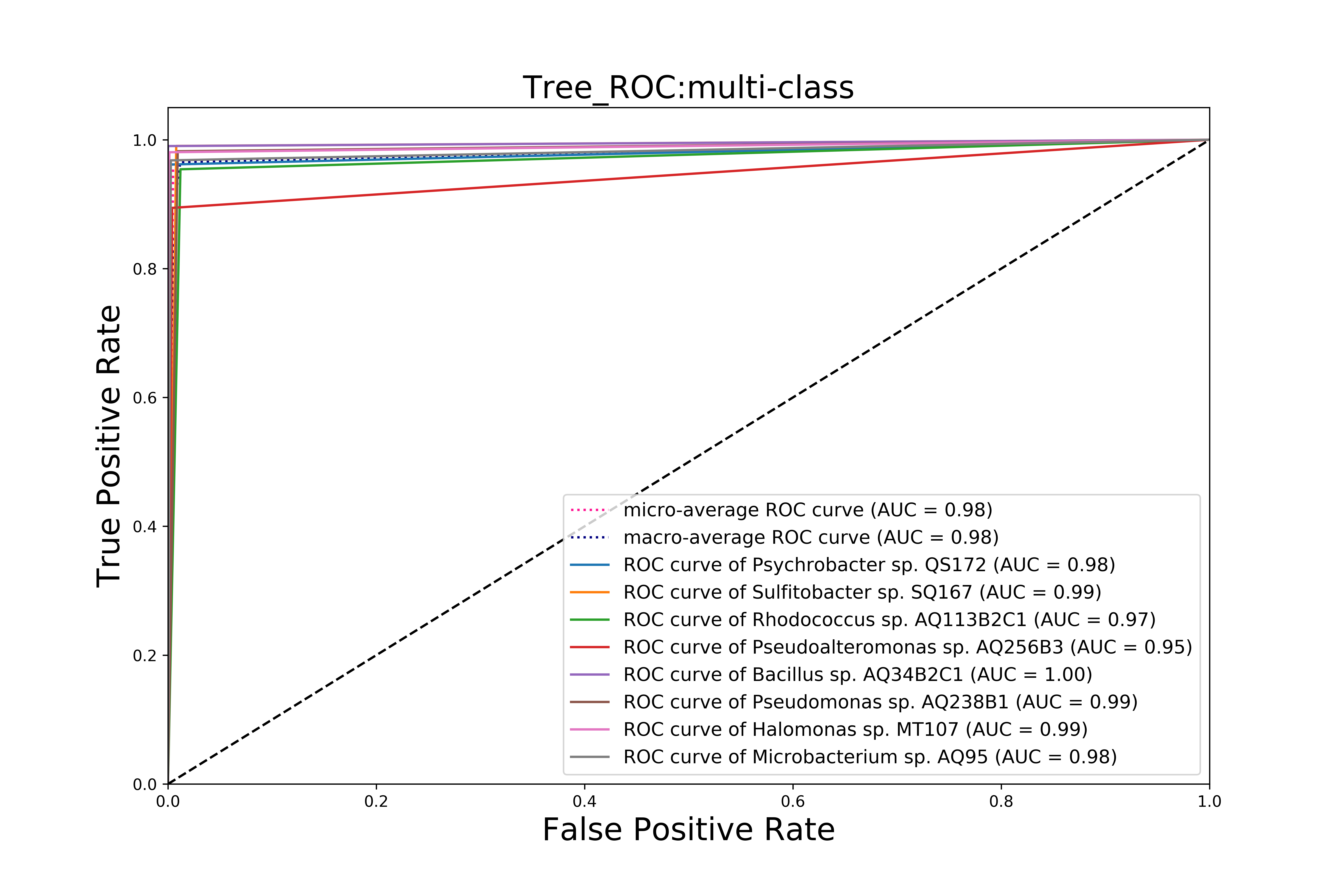


**Fig.S1** (a) Results of prediction of eight bacterial species achieved by the Alexnet taxonomic model. (b) Sensitivity and specificity achieved by the AlexNet taxonomic model.

B

A


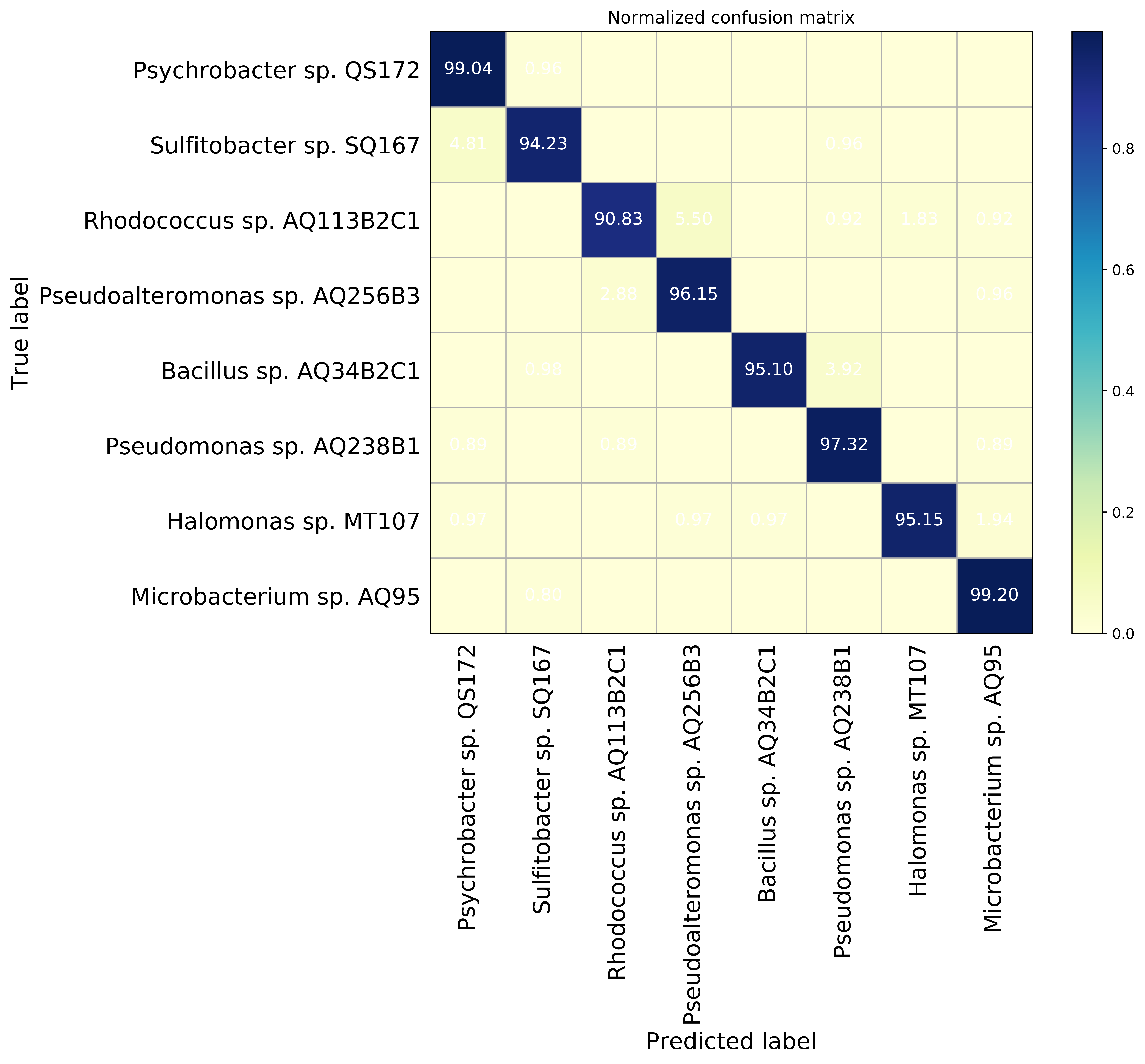

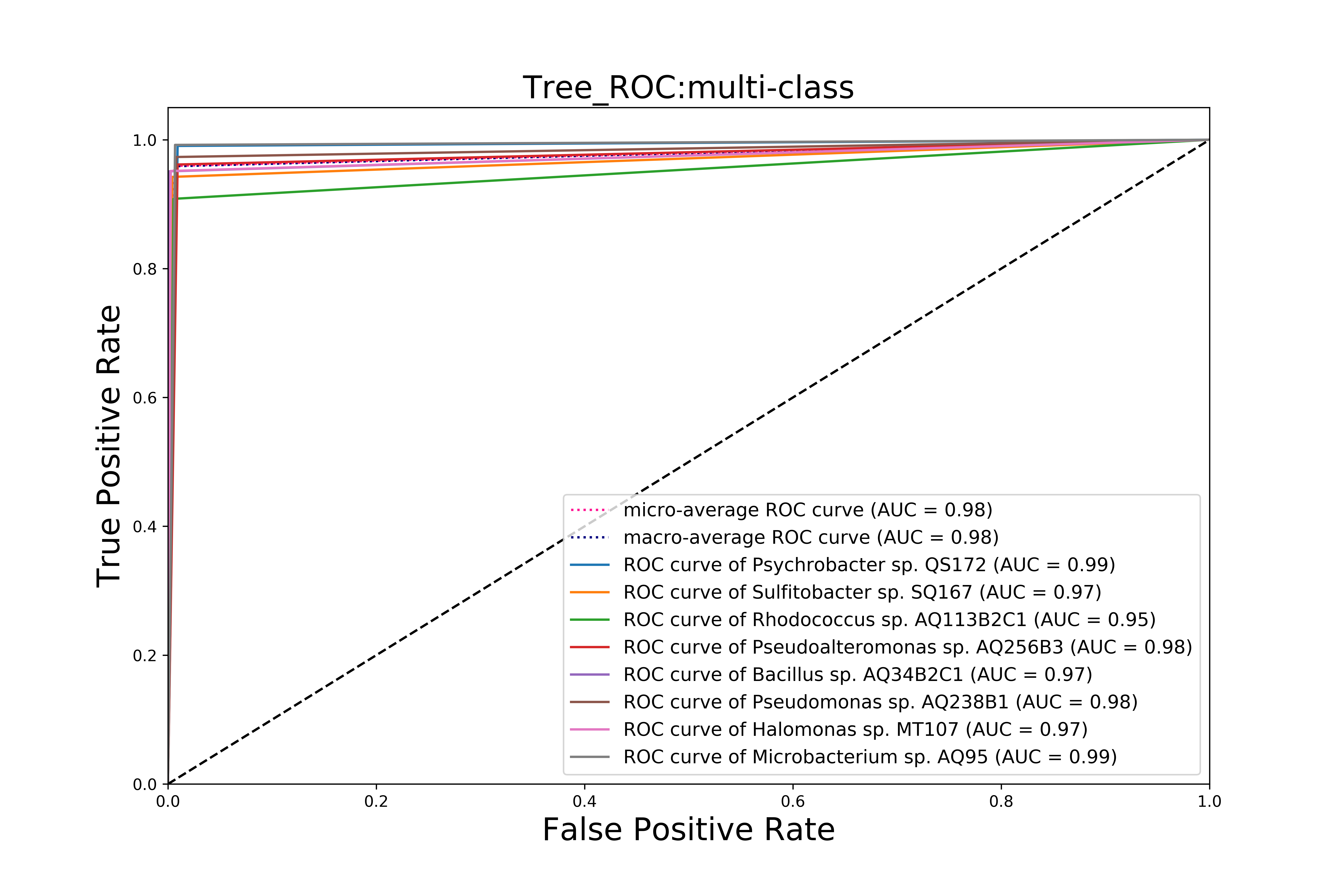


**Fig.S2** (a) Results of prediction of eight bacterial species achieved by the ResNet taxonomic model. (b) Sensitivity and specificity achieved by the ResNet taxonomic model.
